# *Cordyceps militaris* Inhibited Angiotensin-Converting Enzyme through Molecular Interaction between Cordycepin and ACE C-Domain

**DOI:** 10.3390/life12091450

**Published:** 2022-09-19

**Authors:** Mónica A. Valdez-Solana, Iván A. Corral-Guerrero, Alfredo Téllez-Valencia, Claudia Avitia-Domínguez, Jorge A. Meza-Velázquez, Atahualpa Guzmán de Casa, Erick Sierra-Campos

**Affiliations:** 1 Facultad de Ciencias Químicas GP, Universidad Juárez del Estado de Durango, Av. Artículo 123 S/N Fracc. Filadelfia, Gómez Palacio 35015, Mexico; 2 Facultad de Medicina y Nutrición, Universidad Juárez del Estado de Durango, Av. Universidad y Fanny Anitúa S/N, Durango 34000, Mexico; 3 Centro de Investigación y Estudios Avanzados del IPN, Departamento de Biotecnología y Bioquímica, Km 9.6 Libramiento Norte Carretera Irapuato-León, Irapuato 36824, Mexico

**Keywords:** angiotensin-converting enzyme, cordycepin, *Cordyceps militaris*, molecular docking

## Abstract

One of the most important therapeutic modalities for the management of hypertension is the inhibition of the angiotensin-converting enzyme (ACE). *Cordyceps militaris* has received substantial attention because to its therapeutic potential and biological value. To gather information about the antihypertensive properties of *C. militaris*, the ACE inhibitory activity was evaluated. An ethanolic extract of the fruiting body of *C. militaris* was obtained, and the extract was separated by UHPLC method with a fluorescence detector for the quantification of cordycepin and adenosine. The ethanolic extract had a considerably higher cordycepin level. Additionally, an in vitro kinetic analysis was carried out to find out how much *C. militaris* extract inhibited ACE. This extract exhibited non-competitive inhibition on ACE. The Ki value of the *C. militaris* extract against ACE was found to be 8.7 µg/mL. To the best of our knowledge, this is the first report of the analysis of a protein cavity together with molecular docking carried out to comprehend the intermolecular interactions between cordycepin and the ACE C-domain, which impact the spatial conformation of the enzyme and reduce its capacity to break down the substrate. According to a molecular docking, hydrogen bonding interactions between the chemicals and the ACE S2’ subsite are primarily responsible for cordycepin inhibition at the ACE C domain. All these findings suggest that *C. militaris* extract are a kind of natural ACE inhibitor, and cordycepin has the potential as an ACE inhibitor.

## 1. Introduction

Cardiovascular disease has always been the leading cause of death in Mexico, even before COVID-19. Cardiovascular disease accounts for 21% of all deaths in the country, diabetes mellitus is the second leading cause of death at 14%, and cardiovascular complications account for 70% of diabetes deaths. The sixth leading cause of death is cerebrovascular accidents, accounting for 5%. In Mexico, the cardiovascular mortality rate per 10,000 population increased from 9.2 to 12.3 per 10,000 population between 2010 and 2019, while the stroke mortality rate remained stable at 2.8. [1]. Hyperlipidemia, hypertension, diabetes, obesity, smoking, and a sedentary lifestyle are some of the etiologies that lead to the development of cardiovascular disease. [2].

Hypertension is Mexico’s largest chronic non-communicable disease public health challenge. Hypertension affects about 30% of the total Mexican population, three-quarters of whom are under the age of 54 (they are still of labor age); 40% of them do not know they have it, but the rest do. Only half of patients take drugs to control their blood pressure (<140/90 mmHg). Cardiovascular risk factors such as hypertension, dyslipidemia, obesity, and diabetes often coexist and magnify each other in the same person in terms of common pathophysiologic pathways [2].

Angiotensin I-converting enzyme (ACE) is an essential, ubiquitous transmembrane zinc metallopeptidase in mammalian tissues that cleaves dipeptides from the C-termini of peptide substrates. As a regulator of the renin–angiotensin–aldosterone (RAAS) system, ACE plays an important role in regulating blood pressure, supporting cardiovascular homeostasis [3]. In the RAAS system, when blood pressure decreases, the kidney releases angiotensin, an enzyme that degrades angiotensinogen, the precursor of angiotensin I, and ACE degrades angiotensin I to produce angiotensin II, which has vasoconstrictor function. Furthermore, ACE increases blood pressure by inactivating vasodilator peptides such as bradykinin and kallidin, and by stimulating the release of aldosterone [4].

The most common drugs used to treat hypertension, heart failure, and diabetes mellitus are ACE inhibitors due to their ability to modulate angiotensin II levels, vasoconstriction, and aldosterone and bradykinin secretion [5]. However, some of the most common side effects of treatment with these synthetic inhibitors are erectile dysfunction, persistent dry cough, angioedema, and congenital malformations [6]. Hypertension studies have confirmed that ACE inhibitors potentiate the antihypertensive effects of diuretics, but the interaction appears to be additive rather than synergistic. The efficacy of inhibitors in hypertensive patients has been reported to be 40–50% when used as monotherapy and 80–90% when combined with diuretics [7]. Therefore, ACE inhibitors are useful in treating hypertension, many of which are derived from foods such as mushrooms.

*Cordyceps militaris* is known as a functional food in China, Southeast Asia, and North America because its fruiting body contains phytochemicals, which are the main bioactive components. *C. militaris* has multiple biological activities such as prosexual, anti-inflammatory, antioxidant/anti-aging, anti-tumor/anti-cancer/anti-leukemic, anti-proliferative, and anti-metastatic [8]. However, there are few studies on the chemical composition and properties of the bioactive metabolites of *C. militaris* grown in Mexico. Previous studies have shown that the outermost fruiting body matter has the highest concentration of bioactive compounds [9,10,11]. The fruiting body is particularly rich in essential amino acids (lysine and threonine). About 70% of the fatty acids in the fruiting body were unsaturated fatty acids, and the most abundant fatty acid was linoleic acid.

It is important to note that the concentrations of nucleosides, polysaccharides, carotenoids, and organic selenium compounds in the fruiting body of *C. militaris* are not uniform due to the large regional variation in growing conditions. Two of the most valuable components of this fungus are the nucleosides adenosine and cordycepin, but their concentrations vary in each part of *C. militaris*. In the body, adenosine has a concentration of 0.06% and cordycepin 0.36%. In the fruiting body, adenosine has a concentration of 0.18% and cordycepin 0.97% [12].

It has been argued that the angiotensin-converting enzyme (ACE) inhibitory activity of fungi, including *C. militaris*, is likely due to components such as ergothioneine and phenolic compounds [13,14]; however, this has not been demonstrated clearly yet. Despite great interest in the cardiac metabolic properties of *C. militaris*, the chemical structure of this important fungal ACE inhibitory component remains unknown. The objective of the current work was to discover phytochemicals from the fruiting bodies of *C. militaris*, improve their extraction, and perform a preliminary assessment of their potential for use in antihypertensive applications by assessing their effects on the kinetics of ACE activity.

## 2. Materials and Methods

### 2.1. Preparation of Cordyceps militaris Ethanolic Extract

The *C. militaris* dry sample was acquired commercially, by a distributor located in Tlaxcala, México. Seven grams of the *C. militaris* fruiting bodies were processed in a conventional blender until a homogeneous powder was obtained, it was suspended in 35 mL of ethanol (TEDIA UN1170 HPLC grade), subsequently the ethanolic extract was passed through an ultrasonic bath at 42 KHz (BRANSONIC 3510R -MTH) for 2 h at room temperature, the samples were filtered and centrifuged at 1500 g for 5 min, and the extract was concentrated in a rotary evaporator (BUCHI R-210) until a final volume of 6 mL was obtained at 175 mbar and 40 °C.

### 2.2. UHPLC-FD Analysis

In the analysis by UHPLC (Thermo DIONEX-UHPLC UltiMate 3000) with fluorescence detector ((DIONEX UltiMate 3000), the ethanolic extract of *C. militaris* were diluted 1:2000 in HPLC-grade ethanol, and were passed through a syringe filter with a pore size of 0.22 µm. (MS PTFE Syringe Filter) before being injected. The ultra-high resolution liquid chromatography assay was carried out on a Hypersil GOLD™ aQ C18 column (100 mm × 2.1 mm particle size 1.9 µm) at a temperature of 30 °C, 3 µL of each sample was loaded by autosampler at 10 °C in a 100 µL loop. Mobile phase was composed for filtered and degassed solution A (water with 0.1% formic acid) and solution B (acetonitrile). The elution gradient was programmed as follows: minute 0–2 (B) 65%, minute 2–30 (B) 90%, minute 30–70 (B) 90%. The total time of the run was 70 min at a flow of 0.1 mL/min, the fluorescence detector (FD) was used at 45 °C, with an excitation wavelength of 270 nm and an emission of 334 nm. The identification of compounds was made by comparing the retention times of sample peaks with those of the standards.

Standard solutions of adenosine (A9251-5G) and cordycepin (C3394-25MG) were purchased from Merck and prepared at 0.5 mg/mL in ethanol and a series of diluted standards were prepared for UHPLC calibration curves. The criterion of a signal-to-noise ratio (S/N) of at least 3:1 was used as the detection limit. The recovery of a standard marker was defined as the ratio of the amount detected to the amount added.

### 2.3. Measurement of ACE Inhibitory Activity

ACE inhibitory activity of each sample was determined [15]. Ten microliters of *C. militaris* extract was incubated with 30 μL of Hip-puryl-L-histidyl-L-leucine (HHL, 12.5 mM in 0.1 M sodium borate buffer) at 37 °C for 10 min. Ethanol was used instead of extract as a blank and control. After the incubation, 10 μL of ACE (peptidyl dipeptide hydrolase from rabbit lung acetone extract) was added and the mixture was incubated at 37 °C for 30 min. The enzymatic reaction was stopped by adding 50 μL of 1N HCl. The hippuric acid generated by the action of ACE on HHL was extracted from the acidified solution into 300 μL of ethyl acetate by vortexing for 15 s. The extract was centrifuged at 1000 g for 5 min at 4 °C. 250 µL aliquots of each ethyl acetate layer were transferred to clean tubes and evaporated by heating at 70 °C in a water bath for 1 h. The hippuric acid was redissolved in 300 μL of distilled water and the amount formed was determined by the absorbance at 228 nm. ACE inhibitory activity (%) was calculated as:

ACE inhibitory activity (%) = [1 − (As − ASB)/(AC − ACB)] × 100(1)

where AS is the absorbance of the sample, ASB is the absorbance of the sample blank, AC is the absorbance of the control, and ACB is the absorbance of the control blank. Enalapril maleate salt (E6888-1G) was purchased from Merck and used as control.

Kinetic parameters, namely, the Michaelis–Menten constant affinity (K_m_) and maximum velocity (V_max_), were derived from appropriate Michaelis–Menten and Lineweaver–Burk plots using Sigmaplot software v12.3 (SYSTAT, Palo Alto, CA, USA).

### 2.4. Molecular Docking between ACE C-Domain and Cordycepin

The structure of cordycepin and adenosine were drawn using the Canonical SMILE format of PubChem (CID 6303 and 60961, respectively) and its 3D conformation was generated using online cheminformatics [16]. LigPrep generated all possible tautomers and states at pH 7.0 using Epik [17] for each compound. The crystal structure of the ACE C-domain with inserted symmetry molecule C-terminus (PDB ID: 6ZPU) was acquired from the Protein Data Bank (PDB). The protein was prepared using the Protein Preparation Wizard to assign bond orders, add hydrogens at pH 7.0, and remove water molecules. Prime was used to complete missing side chains and loops. Finally, a restrained minimization was performed using the default constraint of 0.30 Å RMSD and the OPLS 2005 force field in order to complete the protein preparation. Molecular docking simulations were performed using the CB DOCK 2.0 induced fit docking module in standard protocol (standard precision) mode [18]. The binding conformations of cordycepin or adenosine were analyzed in order to identify the important interactions with the cavity residues of ACE C-domain with PLIP [19].

### 2.5. Statistical Analysis

For evaluation of the results, all the data were presented as mean ± standard deviation (SD) of three independent determinations.

## 3. Results

### 3.1. UHPLC Analysis

*C. militaris* has become a valuable substitute for *Cordyceps sinensis* because it can be grown in a variety of media and can be kept under laboratory conditions more easily than *C. sinensis* [20]. However, the concentrations of adenosine and cordycepin vary depending on the culture media used and there is high-cordycepin-yielding strains [21]. As a result, a validated method for separating and identifying the main metabolites present in the extract of *C. militaris* in México is required.

**Figure 1:**
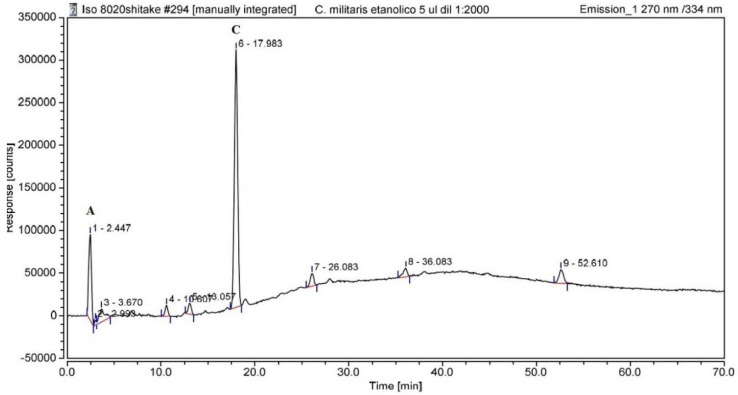
The UHPLC elution pattern of *C. militaris* extract. Peaks were: (1) Adenosine, (2–5 and 7–9) unknown peaks, and (6) cordycepin. Emission wavelength 270 nm—excitation 334 nm. A, adenosine and C, cordycepin.

Under our test conditions, it was possible to obtain a high signal for nucleosides. However, a gradient condition and long run times were needed to facilitate the separation of the metabolites and maintain the symmetry of each peak. In Figure 1 shows a representative UHPLC-FD chromatogram of the *C. militaris* extract. A total of nine peaks were observed in *C. militaris* fruiting bodies’ ethanolic extract. The main identified bioactive constituents were adenosine (A) and cordycepin (C). The detection limit was 0.32 ng/mL for adenosine and 0.15 ng/mL for cordycepin. The recoveries were 98.67% for adenosine, and 99.77% for cordycepin. Calibration curves were linear over a large concentration range of 1.25–150 μg/mL for cordycepin and 6.5–90 μg/mL for adenosine. The calibration curves of standards were as follows: y = 71387x − 10254, R^2^ = 0.9987 for cordycepin; y = 35018x +460.6, R^2^ = 0.9979 for adenosine. The *C. militaris* extract had 2.9 ± 0.3 mg/g adenosine and 16.84 ± 1.7 mg/g cordycepin. It is important to note that in the chromatogram of the ethanolic extract it was not possible to identify any flavonoids or polyphenols. Furthermore, the antioxidant capacity of the extract was negligible with the ABTS and DPPH tests (data not shown). Therefore, the remaining peaks with different retention times were not identified but could be alkaloids or other nucleoside analogues. Consequently, additional liquid chromatography studies coupled with mass detectors are required to accurately identify all the components present in ethanolic extracts of *C. militaris*.

The results are consistent with previous reports. However, a review of the relevant literature revealed great variability in cordycepin content in ascomata and mycelia of *C. militaris*. Despite intensive efforts to improve cordycepin production by *C. militaris*, altering culture conditions or supplementing the substrate with different nutrients resulted in consistently high cordycepin concentrations in the harvested biomass. Consequently, large fluctuations are commonly observed in cordycepin values measured either in mycelia generated from liquid fermentation (i.e., from 30 mg L^−1^ up to 8570 mg L^−1^) or in cultivated ascomata (i.e., from 0.6 mg g^−1^ up to 77.4 mg g^−1^) [22]. This variability is due to many factors, one of the most important of which is the strain used. As previously mentioned, the genetic makeup of *C. militaris* appears to have a profound effect on both the ability to form ascomata and, most importantly, the cordycepin biosynthetic capacity of individual strains. Furthermore, substrate composition can also have a significant impact on cordycepin production and the analytical methodology applied and the cordycepin extraction protocol used to quantify cordycepin also give very different values that are difficult to interpret and are related to the fungi and culture conditions used [22].

### 3.2. Kinetics of ACE Inhibition

We speculate that *C. militaris* may regulate antioxidant defenses through a mechanism involving ACE pathway. Therefore, we measured the inhibitory activity of *C. militaris* extract and cordycepin on ACE.

**Figure 2:**
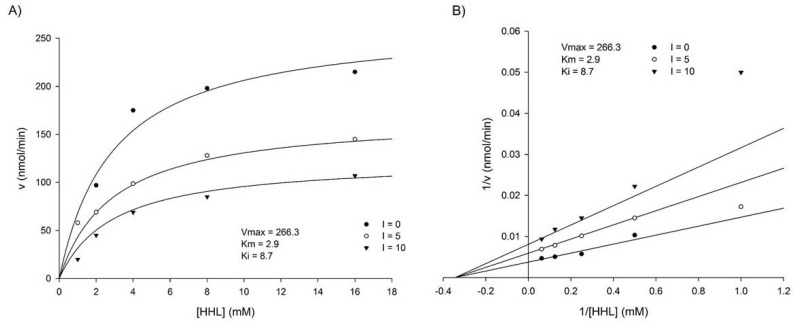
(**A**) Initial velocities for the inhibition of the ACE activity by *C. militaris* extract vs. substrate concentration. (**B**) The Lineweaver–Burk plot, indicating the type of inhibition for *C. militaris* extract.

The kinetics parameters of this study are consistent with that of [23], which reported the apparent Km and kcat values for purified testis ACE determined from a double reciprocal plot were 3.0 mM and 195.7 s^−1^, respectively. As shown in Figure 2A, ACE activities were reduced by the *C. militaris* extract in a dose-dependent manner and increased with the substrate in a concentration-dependent manner. Based on the Michaelis–Menten plot, *C. militaris* extract showed a decrease in V_max_ value as compared to the uninhibited reaction. This effect may be attributed to the presence of potential ACE inhibitors, which can act as non-competitive inhibitors of ACE. We examined the mechanism of this inhibitory effect using a Lineweaver–Burk double reciprocal plot of reaction rate versus HHL concentration with different concentrations of the *C. militaris* extract (Figure 2B). The results indicate that the y-intercept and x-intercept depend on the concentration of the *C. militaris* extract. These changes in the apparent V_max_ and K_m_ indicate that the *C. militaris* extract inhibited ACE by a noncompetitive mechanism. The equilibrium constant (K_i_) for inhibitor binding with free ACE, determined by linear regression of the apparent 1/ V_max_ versus *C. militaris* extract concentration, was 8.7 μg/mL. Therefore, the extract has a stronger affinity with ACE-HHL complex.

Our findings support the use of *C. militaris* in folk medicine for several cardiovascular diseases. Cordycepin inhibited platelet-derived growth factor-BB (PDGF-BB)-induced RASMCs migration and proliferation in a dose-dependent manner [24]. Moreover, cordycepin from *C. militaris* has been shown to lower triglycerides, total cholesterol, low-density lipoprotein (LDL), and very low-density lipoprotein (VLDL) levels, in an animal model of hyperlipidemia. Cordycepin showed the features of an AMPK activator and an inhibitor of lipoprotein and hepatic lipase [25]. Other studies suggests that cordycepin decreased lipid accumulation via activating AMPK and regulating mitochondrial function in oleic acid (OA)-induced mouse FL83B hepatocytes while the anti-fatty liver effect of cordycepin on regulating lipogenesis and fatty beta-oxidation was abolished by AMPK inhibitor compound C treatment. These results suggested that cordycepin attenuated lipid accumulation by promoting beta-oxidation and may be developed as a functional food/natural medicine for fatty liver suppression [26].

### 3.3. Molecular Docking between ACE C-Domain and Cordycepin

ACE is a two-domain dipeptidyl carboxypeptidase directly involved in the regulation of blood pressure by hydrolyzing angiotensin I to produce angiotensin II. At the same time, ACE hydrolyzes other substrates such as the vasodilatory peptide bradykinin and the anti-inflammatory peptide N-Acetyl-Seryl-Aspartyl-Proline (N-acetyl-SDKP) [27]. In this sense, ACE inhibitors are bioactive substances that are potentially used as drugs to treat or prevent hypertension, heart failure, myocardial infarction, and other important diseases. It is well known that the lack of selectivity of ACE inhibitors is the cause of the adverse side effects due to progressive increments of BK in the patients [28]. ACE has two active sites, N- and C-terminal, with different affinities for different substrates. Both ACE domains are very similar: with an elliptical structure crossed by a long and deep cleft in the active site and with a predominance of -helices. The binding cavity is capped and contains a Zn^2+^ ion in the center; four subsites, designated S_2_, S_1_, S_1_′, and S_2_′, are located on either side of the central ion [27]. Selective ACE inhibitors may be able to block the activities of either of the two homologous domains, nACE or cACE. Although both domains hydrolyze BK [29], their biological functions are different since hydrolysis of angiotensin I depends exclusively on cACE [30] and the anti-inflammatory peptide N-acetyl-SDKP is hydrolyzed by nACE [31]. Therefore, selective cACE inhibitors are expected to reduce angiotensin II production while BK is normally hydrolyzed, resulting in BK-mediated angioedema reduction [32]. Consequently, an attenuation of vasodilator side effects can be achieved when blood pressure is controlled by selective inhibition of cACE [33]. On the other hand, selective inhibition of nACE increases N-acetyl SDKP levels, resulting in desirable effects in the prevention of cardiovascular and renal inflammation and fibrosis [34]. Selective inhibition of nACE does not prevent the action of the other cACE domain, which contributes to normal blood pressure regulation.

**Figure 3:**
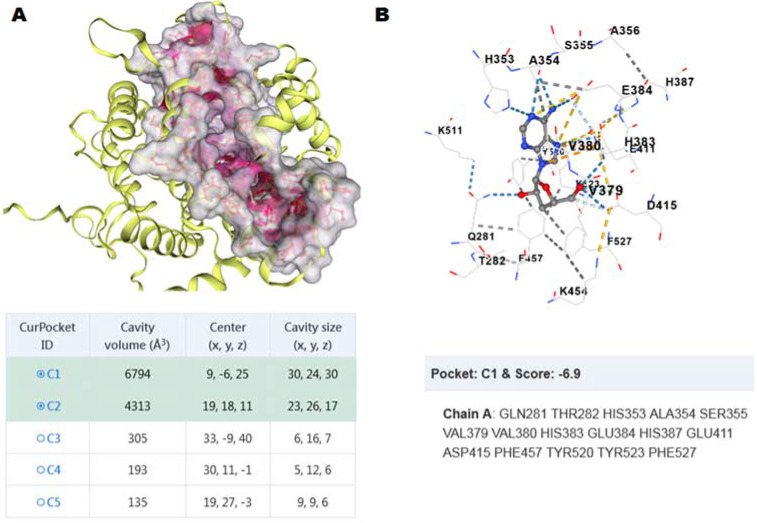
(**A**) Docked pose of the cordycepin inside the cavity 1 of Native cACE (PDB ID: 6ZPU). (**B**) Hydrogen bonds are represented as blue dotted lines, hydrophobic interactions are represented as gray dotted lines, and ionic interactions are represented as yellow dotted lines.

A simple method for selecting inhibitors is in silico analysis. Molecular docking simulations have been widely used to predict protein and ligand binding affinities. The technique has been successfully used to screen active compounds of herbal samples with pharmacological effects [35]. In silico analyses showed that spermidine derivatives identified in the pulp of Lulo (*Solanum quitoense* Lam.) had potent ACE-I inhibitory activity [36]. Thus, molecular docking was used in order to understand the binding mode of the most active compound, cordycepin, inside the main cavity (volume of 6794 Å^3^) of ACE C-domain generated by CB Dock 2.0, which is formed for sixteen amino acid residues (Figure 3A). We used a standard protocol to perform induced fit docking and all the parameters were set to their defaults, which generated 5 binding poses for cordycepin. All five poses were analyzed for their docking score and their bonding and non-bonding interactions. One of the poses showed a docking score of −6.9 kcal/mol (Figure 3B) and this pose was selected and further interactions were examined by PLIP. Cordycepin form six hydrogen bond (H-bond) interactions with ACE pocket. The pyridine ring of cordycepin formed two H-bonds with ALA354 and Glu384 and NH_2_ group formed an H-bond interaction between the H atom of the NH group of pyridine and HIS353. Another H-bond interaction was observed between the oxygen atom of the hydroxymethyl and residue ASP415. Similarly, the C atom of the imidazole moiety formed a hydrophobic interaction with PHE527. Additionally, the 3-ol of cordycepin formed an H-bond with GLN281. It is interesting to mention that H383, D415, and F527 are part of S_2_′ subsite, while H353 and A354 are part of S_1_′ subsite and E384 interact with catalytic Zn^2+^.

These results agree with the kinetic data of the ethanolic extract, since if cordycepin binds near or in the catalytic site of the enzyme, the Vmax value decreases, but it does not affect the active site because its structure is not analogous to the substrate (peptidic nature). In addition, there is the possibility that the same cordycepin-binding site may recognize other nucleotides such as adenosine. Molecular modeling between adenosine and ACE C-domain indicates that they can form a stable complex with a binding energy of −7.7 kcal/mol. However, the cordycepin/adenosine ratio is approximately 6-fold, making it more likely that the major metabolite for the inhibitory activity of the extract is cordycepin.

## 4. Conclusions

The kinetics of ACE inhibition by a *C. militaris* extract were examined in this work. With a Ki of 8.7 μg/mL, the results show that this extract decreased ACE activity in a dose-dependent manner. According to a Lineweaver–Burk double reciprocal plot, the extract of *C. militaris* acted as a noncompetitive ACE inhibitor. The bioactive elements of the *C. militaris* extract may be nucleosides, such as adenosine and cordycepin, according to UHPLC-FD. Cordycepin was discovered to have the lowest dock score during docking study, suggesting the strongest interaction with the ACE C-terminal S2′ subsite. In conclusion, these results provide a foundation for the potential use of *C. militaris* in pharmaceutical and nutrition products. In addition, cordycepin could be developed as a new antihypertensive agent. However, more research into these potentially therapeutic effects is needed using animal models such as the spontaneously hypertensive rat.
